# Transcriptome dataset of light-dependent expression in the early onset retinal degeneration model, *Mcoln1^−/−^* mouse

**DOI:** 10.1016/j.dib.2023.109659

**Published:** 2023-10-15

**Authors:** Rebecca Cistulli, Jonathan G. Miller, Ray A. Enke, Marquis T. Walker

**Affiliations:** James Madison University, Department of Biology, United States

**Keywords:** RNA-seq, Retina, Photoreceptor, Mucolipidosis type IV (MLIV), Retinal degenerative disorders (RDDs)

## Abstract

Retinal degenerative diseases (RDDs) are a diverse group of retinal disorders that cause visual impairment. While RDD prevalence is high, little is known about the molecular mechanisms underlying the pathogenesis within many of these disorders. Here we use transcriptome analysis to elucidate the molecular mechanisms that drive early onset photoreceptor neuron function loss in the mouse model of the RDD Mucolipidosis type IV (MLIV). MLIV is a lysosomal storage disorder resulting from loss of function mutations in the *MCOLN1* gene. *MCOLN1* encodes a lysosomal cation channel, the transient receptor potential channel mucolipin 1 (Trpml1). To identify changes in gene expression during onset in MLIV we used a genetic mouse model (*Mcoln1^−/−^*) which recapitulates clinical attributes of the human disease. We conducted transcriptome analysis in 6-week old control and *Mcoln1^−/−^* mice under normal 12:12 light cycle as well as low and high light stress conditions. These data will be valuable to the vision research community for identifying differentially expressed in early onset MLIV potentially leading to new insights into the pathophysiology of this RDD. Raw FASTQ files and processed counts files for the RNA-seq libraries are deposited in the NCBI Sequence Read Archive (SRA) and have been assigned BioProject accession PRJNA1002601 [1].

Specifications Table

**Table:** 

Subject	Neuroscience: Sensory systems
Specific subject area	Transcriptome analysis of a retinal degenerative disease
Data format	Raw data: fastq sequencing files Secondary data: counts table of reads aligned to coding exons
Type of data	Table, Image, Chart, Graph, Figure
Data collection	PolyA+ RNA sequencing, Illumina HiSeq 4000 2×150bp PE reads
Data source location	• Institution: James Madison University • City/Town/Region: Harrisonburg, VA • Country: USA
Data accessibility	Repository name: NCBI SRA Data identification number: PRJNA1002601 Direct URL to data: https://www.ncbi.nlm.nih.gov/bioproject/PRJNA1002601/ Instructions for accessing these data: download raw and/or processed files

## 1 Value of the Data

• These datasets will be valuable to the vision research community for characterizing global changes in mouse retinal gene expression associated with the *Mcoln1^−/−^* mutation as well as differing light exposure regiments.
• These transcriptome datasets may be used to identify differentially expressed genes associated with the retinal degenerative disease Mucolipidosis type IV (MLIV).
• This bioinformatics analysis pipeline applied in this study exclusively uses open access tools to ensure reproducibility of robust eukaryotic transcriptome analysis.
• All data from this project is publicly available via the journal website and the NCBI SRA database

## 2 Data Description

**Fig. 1:**
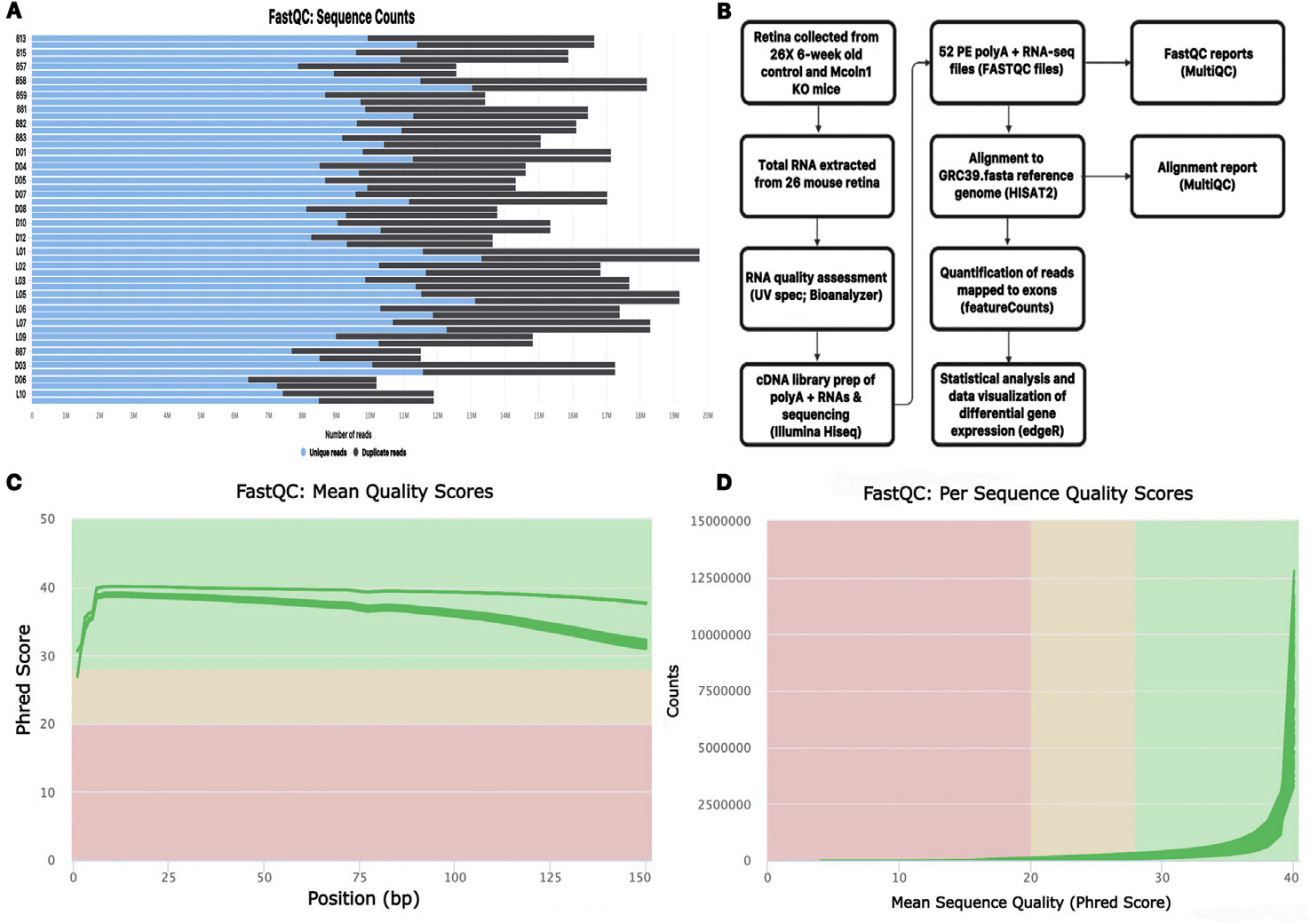
Overview of sequence quality control and workflow of data analysis. (A) Sequence counts histogram for each sample illustrating duplicate and unique reads. (B) Pipeline of tissue collection from mice retinas to bioinformatic tools used for downstream data analysis. (C) Per sequence Phred scores showing the high quality of the sequenced reads with most scores > 30. (D) Per base Phred scores showing the high quality of sequenced base pairs with almost all samples being > 30.

Mucolipidosis type IV (MLIV), a prototypical retinal degenerative disorder, is an autosomal recessive lysosomal storage disorder (LSD) caused by mutations in the *MCOLN1* gene [2]. *MCOLN1* encodes the mucolipin-1 protein, a member of the superfamily of transient receptor potential (TRP) cation channels and is commonly identified as Trpml1 [2]. This study aims to identify molecular targets that drive the progressive PR neuron function loss in a mouse model for MLIV. In this study we analyze expression in 6-week old retinas because preliminary evidence in our lab demonstrates that *Mcoln1^−/−^* mutant mice at 4-weeks old have wild-type light responses, but beginning at 6-weeks of age these mice have significant physiological defects in retinal PR neuron light responses, prior to retinal degeneration. Here we have applied RNA-sequencing (RNA-seq) transcriptome analysis to 6-week old control and *Mcoln1^−/−^* mouse retinas under normal light and light stress conditions as a tool to better understand disease progression of the RDD MLIV. While several previous studies have measured global transcription in *Mcoln1^−/−^* brain tissue, this is the first public data set detailing global expression in *Mcoln1^−/−^* retina [3,4]. These analyses were conducted using Illumina mRNA-seq in tandem with a bioinformatics pipeline exclusively using open access tools to ensure sequence quality and robust eukaryotic transcriptome analysis (Fig. 1). The experiment described here is part of an ongoing NSF-funded project hosted by the Cold Spring Harbor Laboratory, DNA Learning Center (CSHL DNALC) and the James Madison University Center for Genome and Metagenome Studies (JMU CGEMS) focused on incorporating RNA-seq analysis into undergraduate education (http://www.rnaseqforthenextgeneration.org).

## 3 Experimental Design, Materials and Methods

### 3.1 Genotyping

The *Mcoln1^−/−^* mouse line has a targeted deletion between exons 2-5 in the *MCOLN1* gene causing a loss of Trpml1 and a disruption in autophagy [5] (Venugopal et al., 2007). In order to maintain the *Mcoln1^−/−^* mouse line and specifically select for knockout and control animals, we conduct routine genotyping using DNA extracted from 1-2 mm tail sections from each mouse in a newly separated group of pups. Tails were lysed using 200μl of DirectPCR Lysis Reagent and 5μl of proteinase K incubated at 55℃ overnight. Protease was heat killed at 85°C for 45 mins. PCR was used to amplify the *MCOLN1* locus and gel electrophoresis was used to visualize band sizes to determine genotypes of individual mice. For this study, homozygous *Mcoln1^−/−^* mice were used for the experimental group and either homozygous wt or *Mcolnl-/MCOLN1* heterozygous mice were used for as the control group.

### 3.2 Light condition experimental set-up

Individual groups of control and *Mcoln1^−/-^* mice were raised for 6 weeks in three separate light conditions: 12:12 light/dark (LD), Constant Dark (CD), or Constant Light (CL) as previously described [6]. Sample size for each condition was 5 control, 4 *Mcoln1^−/-^* in LD, 4 control, 4 *Mcoln1^−/-^* in CL, 5 control, 4 *Mcoln1^−/-^* in CD. Once females were determined to be pregnant, they were transferred to one of the light conditions. Upon weening (∼3 weeks), mothers were removed and pups continued to live in their respective light conditions and routine weekly check ups were completed as per the Institutional Animal Care & Use Committee (IACUC) protocol. After 6 weeks, animals were euthanized and both eyes were harvested for retina collection. Both retinas from each individual were pooled for a single total RNA extraction and downstream transcriptome analysis.

### 3.3 RNA-sequence analysis of retinal RNA

6-week-old mice were euthanized via CO₂ and their eyes were enucleated and dissected to obtain whole retina from each eye. Retinas were immediately frozen using liquid nitrogen and stored at -80°C until total RNA extraction was performed using a Qiagen RNeasy kit following the manufacturer's protocol with an on column DNase I treatment as previously described [7]. RNAs quality and quantity was initially assessed using UV spectrophotometer and Agilent 2100 Bioanalyzer analysis. Samples chosen for characterization of global mRNA expression were submitted to Azenta Life Sciences (Chelmsford, Massachusetts). Illumina stranded TrueSeq cDNA libraries were constructed using poly dT enrichment for each sample according to the manufacturer's protocol. The resulting average size of the cDNA libraries was approximately 300 bp. Libraries for the 26 cDNA samples were sequenced using the Illumina HiSeq 4000 platform yielding 10.2-19.8 million 150 bp paired end (PE) sequence reads per sample (Table 1). Raw sequence data was returned and analyzed using a previously established open access bioinformatics package (Fig. 1; [8]).

**Table 1:** RNA-seq samples, read metrics, and public SRA accessions.

Sample name	Sequencer	Treatment group	# of reads (M)	Uniquely mapped reads (M)	Uniquely mapped reads %	SRA accession #
815	Illumina Hiseq 4000 2×150	Control Light/Dark	15.9	13.7	86.2	SRR25533718
881	Illumina Hiseq 4000 2×150	Control Light/Dark	16.5	14.2	86.1	SRR25533717
882	Illumina Hiseq 4000 2×150	Control Light/Dark	16.1	13.9	86.3	SRR25533715
883	Illumina Hiseq 4000 2×150	Control Light/Dark	15.1	13	86.1	SRR25533714
887	Illumina Hiseq 4000 2×150	Control Light/Dark	11.5	9.6	83.5	SRR25533719
813	Illumina Hiseq 4000 2×150	Knockout Light/Dark	16.6	14.2	85.5	SRR25533713
857	Illumina Hiseq 4000 2×150	Knockout Light/Dark	12.6	10.9	86.5	SRR25533712
858	Illumina Hiseq 4000 2×150	Knockout Light/Dark	18.2	14.9	81.9	SRR25533711
859	Illumina Hiseq 4000 2×150	Knockout Light/Dark	13.4	11.5	85.8	SRR25533710
L03	Illumina Hiseq 4000 2×150	Control Light	17.7	14.5	81.9	SRR25533728
L06	Illumina Hiseq 4000 2×150	Control Light	17.4	14.6	83.9	SRR25533727
L09	Illumina Hiseq 4000 2×150	Control Light	14.8	12.7	85.8	SRR25533716
L10	Illumina Hiseq 4000 2×150	Control Light	11.9	9.9	83.2	SRR25533709
L01	Illumina Hiseq 4000 2×150	Knockout Light	19.8	16.7	84.3	SRR25533708
L02	Illumina Hiseq 4000 2×150	Knockout Light	16.8	14.2	84.5	SRR25533707
L05	Illumina Hiseq 4000 2×150	Knockout Light	19.2	16.3	84.9	SRR25533706
L07	Illumina Hiseq 4000 2×150	Knockout Light	18.3	15.4	84.2	SRR25533705
D04	Illumina Hiseq 4000 2×150	Control Dark	14.6	12.3	84.3	SRR25533704
D07	Illumina Hiseq 4000 2×150	Control Dark	17	14.4	84.7	SRR25533703
D08	Illumina Hiseq 4000 2×150	Control Dark	13.8	11.6	84.1	SRR25533726
D10	Illumina Hiseq 4000 2×150	Control Dark	15.3	13.1	85.6	SRR25533725
D12	Illumina Hiseq 4000 2×150	Control Dark	13.6	11.6	85.3	SRR25533724
D01	Illumina Hiseq 4000 2×150	Knockout Dark	17.1	14.1	82.5	SRR25533721
D03	Illumina Hiseq 4000 2×150	Knockout Dark	17.3	14.4	83.2	SRR25533723
D05	Illumina Hiseq 4000 2×150	Knockout Dark	14.3	12.5	87.4	SRR25533720
D06	Illumina Hiseq 4000 2×150	Knockout Dark	10.2	8.6	84.3	SRR25533722

### 3.4 Bioinformatics analysis and sequence alignment QC

**Fig. 2:**
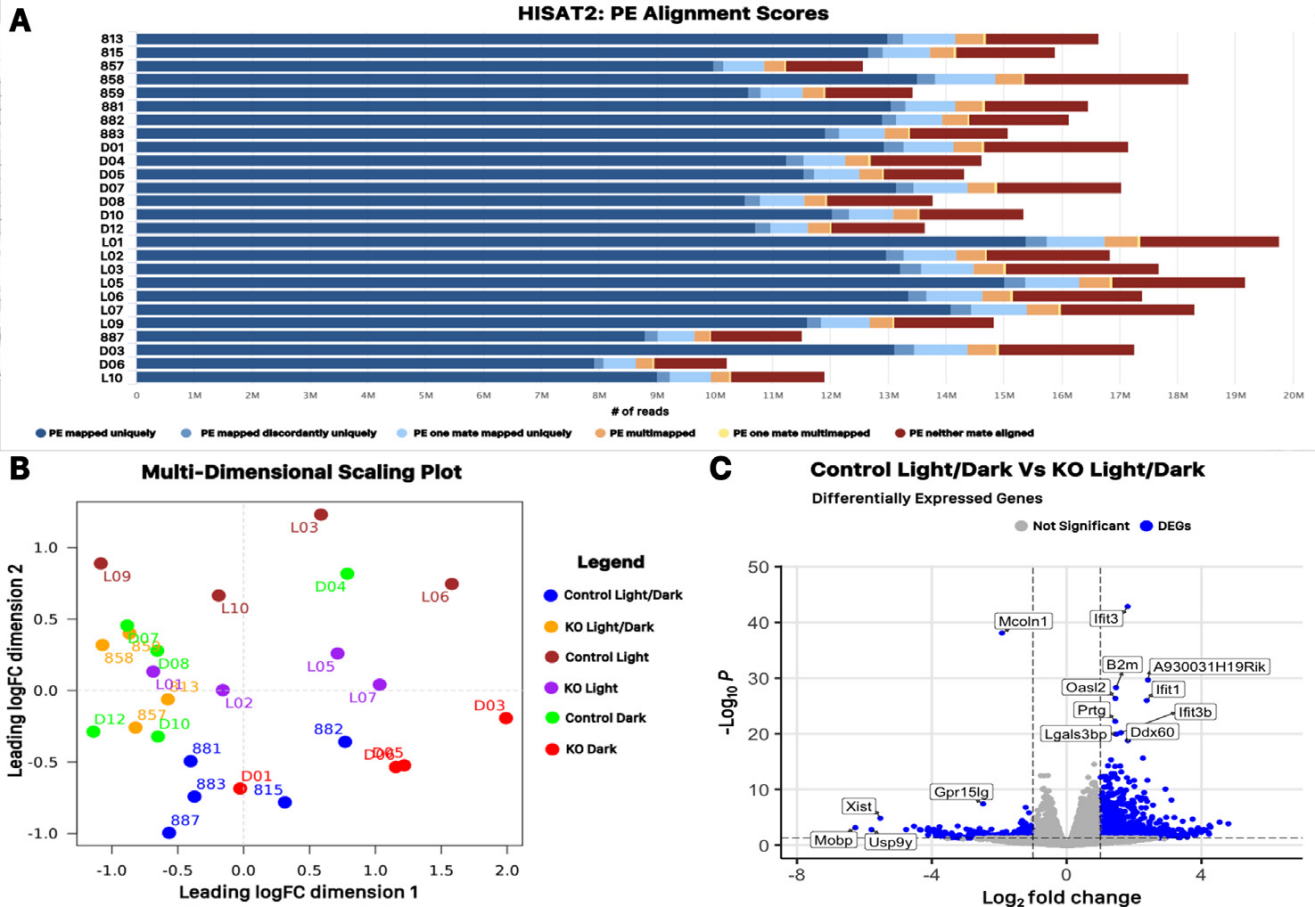
Overview of sequence alignment and data dispersion. (A) Sequence alignment histogram showing the number of HISAT2 paired ends (PE) properly or improperly aligned to the mouse mm39 reference genome. (B) Multidimensional scaling (MDS) plot illustrating the dispersion of each of the individual samples according to their variance in the two dimensions. (C) Volcano plot displaying up-regulated and down-regulated genes in the KO Light/Dark treatment compared to the Control Light/Dark treatment. These differentially expressed genes (DEGs) are plotted with respect to the log2 fold Change and -log10 P Values.

Fig. 1b outlines our project overview including the computational pipeline applied to our raw sequencing data. QC of sequencing reads in the 52 FASTQ files was measured using FastQC and MultiQC analyses (see Code Availability 1-2). Fig. 1c-d shows that all FASTQ sequencing files have an average per sequence and per base Phred score >28 respectively, a common threshold demonstrating high quality base calls. High quality sequence reads were aligned to the mouse GRCm39 reference genome using the HISAT2 splice-aware aligner (see Code Availability 3; [9]). A combined data visualization for HISAT2 alignments was generated using the MultiQC program (see Code Availability 2). An average of 13.2 million reads per sample mapped uniquely to the GRCm39 genome ranging from 82.3% to 90.3% of total input reads (Table 1; Fig. 2a). Collectively, these data show the high quality sequencing and mapping of RNA-seq reads and demonstrate that they meet the criteria for downstream transcriptome differential gene expression analysis.

### 3.5 Differential gene expression analysis

HISAT2 alignments were used as input files for featureCounts software analysis to count reads in each sample mapping to coding exons annotated in GRCm39 mouse genome (see Code Availability 4, [10]. Supplementary Table 1 is the subsequent gene count table used as the input file for the edgeR statistical package to determine differential gene expression between samples (see Code Availability 5; [11]. The edgeR software package was also used to generate a multidimensional scaling (MDS) plot showing the variance between distinct sample groups as well as similarity within sample replicates for all 26 samples (Fig. 2b). To specifically highlight the utility of this dataset for studying transcriptome variance associated with MLIV RDD retina, differential gene expression output tables from control and *Mcoln1-/-* 12:12 light/dark retinal samples were used to create a volcano plot representing significant differentially expressed genes (DEGs) between these two samples groups in terms of -log10(false discovery rate) versus log2(fold change). As a proof of principle, this analysis demonstrates that the mutated *MCOLN1* gene is one of the top DEGs identified between these sample groups (Fig. 2c).

### 3.6 Code availability

The following open access programs and versions were used for quality control, data analysis and visualization as described in the main text:

• FastQC, version 0.11.5 was used was used within CyVerse Discovery Environment for quality analysis of raw FASTQ sequencing data: https://www.bioinformatics.babraham.ac.uk/projects/fastqc/.
• MultiQC, version 1.11 was used was used within CyVerse Discovery Environment to aggregate FastQC and HISAT2 data outputs: https://multiqc.info.
• HISAT2-index-align-2.1 was used was used within CyVerse Discovery Environment to index and align FASTQ reads to the mouse GRC.m39 genome: http://daehwankimlab.github.io/hisat2/.
• featureCounts, version 1.6.0 was used was used within CyVerse Discovery Environment to assign sequence reads to exonic features of the mouse GCR.m39.109 genome: https://subread.sourceforge.net/featureCounts.html#:∼:text=featureCounts%20is%20a%20highly%20efficient,and%20genomic%20DNA%2Dseq%20reads.
• edgeR, version 2.0 was used was used within CyVerse Discovery Environment to quantify and visualize differentially expressed transcripts across all treatments: https://bioconductor.org/packages/release/bioc/html/edgeR.html.

All code and scripts used for quality assessment and data analysis in this study are available at: https://github.com/JonathanGM70/Mcoln1-.

## 4 Technical Validation

### 4.1 RNA quantity and quality

Quantity and quality of extracted RNA was measured using UV spectrophotometry as well as an Agilent 2100 Bioanalyzer to calculate an RNA integrity number (RIN). RIN outputs the RNA quality of the samples on a scale of 1 to 10 (10 being the highest quality). RNA samples with a RIN ≥8 were used for downstream sequencing analysis.

### 4.2 Sequencing read and alignment quality

FastQC and MultiQC software were used to demonstrate that the mean Phred quality scores of sequencing reads are within the acceptable range for downstream data analysis (Fig. 1c-d). FASTQ files contained 10.2-19.8 high quality reads per sample (Fig. 1a; Table 1). 79.56% - 87.41% of these high-quality raw reads were successfully mapped to the mouse GRCm39 genome assembly (Fig. 2a, Table 1).

### 4.3 Usage notes

The bioinformatics pipeline applied to our transcriptome data set outlined in Fig. 1b was obtained using a collection of open access software tools. These analyses however, are interchangeable with many other currently available tools for achieving different experimental outcomes. Our high quality raw FASTQ data can be aligned to any available mouse reference genome or transcriptome, including the most recent GRCm39 reference assembly using a variety of freely available aligners. In this study we used HISAT2 to align high quality reads to a reference genome. HISAT2 is a splice-aware aligner capable of mapping reads that span annotated exon/intron junction in a reference genome. This tool has been validated for use in determining differential gene expression in eukaryotic RNA-seq projects [12]. Our data set may also be used with other transcriptome analysis tools such as Kallisto, which produces pseudoalignment rather than alignment output files.[13]. This alignment-free analysis offers a much faster and smaller data footprint alternative to traditional RNA-seq pipelines that may be suitable for certain situations where time and data storage may be limited (i.e. for course-based research implementations). Here our gene quantification and differential gene expression analysis was carried out using the featureCounts and edgeR software suites, however other publicly available packages such as StringTie may also be used for similar analysis [12]. Critically, data presented in Fig. 1, Fig. 2 demonstrates the high quality of our sequencing reads, read alignment and precision of sampling respectively making this data set compatible with any RNA-seq tools currently available as well as tools that become available in the future.

Our data set will be useful for a variety of studies investigating neuronal degeneration. Specifically, these data can be leveraged to study the underlying mechanisms associated with retinal degeneration in MLIV. In this study we expose control and *Mcoln1^−/−^* mice to previously established standard light and light stress environments [6]. This experimental design is optimal for determining the role of lysosomal storage defects in the onset and progression of PR neuron dysfunction associated with MLIV, but also has several other potential applications. These data can be used in conjunction with previously published transcriptome data sets investigating *Mcoln1^−/−^* mutant mouse and human brain tissue to study wider syndromic pathology of MLIV [3,4]. Alternatively, our novel transcriptome data set can be combined with other available retinal genomics and epigenomics data sets to extend and gain new insight into public domain omics data [14], [15], [16].

Several considerations must be taken into account when using these data for downstream analysis. RNAs were extracted from whole mouse retinas without any further enrichment. Therefore, the resulting analysis will be representative of heterogeneous mixtures of retinal neurons and other cell types found within these tissues. Additionally, cDNA libraries were transcribed using a poly dT primer and the resulting data set is representative of only polyadenylated transcripts. This targeted analysis leaves out any non-polyadenylated transcripts. Lastly, despite the quantity of sequence reads per sample in this study is below the threshold for rare isoform analysis, there are sufficient reads in this dataset to analyze abundant alternative transcript isoforms as previously demonstrated in Gage et al., 2022 [17]. Taking these considerations into account, these data will be a useful resource for robust and accurate analysis of polyadenylated transcriptional networks in the mouse *Mcoln1^−/−^* mutant.

## Limitations

Note: we have included experimental limitations in the last paragraph of the Technical Validation section.

## Ethics Statement

All animal experiments were conducted under the provisions of the National Institutes of Health guide for the care and use of laboratory animals and are in accordance with approved JMU IACUC protocol #23-3903.

## CRediT authorship contribution statement

**Rebecca Cistulli:** Investigation, Resources, Software, Writing – review & editing. **Jonathan G. Miller:** Formal analysis, Software, Visualization, Data curation, Writing – review & editing. **Ray A. Enke:** Conceptualization, Methodology, Writing – review & editing, Supervision. **Marquis T. Walker:** Conceptualization, Methodology, Writing – review & editing, Supervision, Project administration, Funding acquisition.

## Data Availability

Characterizing the effects of light on the early onset photoreceptor function loss in Mucolipidosis type IV (Original data) (NCBI SRA).

## Appendix Supplementary materials

Image, application 1
